# Bromide of Camphor

**Published:** 1874-11

**Authors:** 


The Action of Mono-Bromide of Camphor.—M. Bourneville gives (*Le Progres Medical*) a series of experiments performed by himself on various animals, to whom he administered various doses of this substance, with a view to ascertain its physiological action. His conclusions are as follows: Bromide of camphor diminishes the number of pulsations of the heart, and determines a contraction of the auricular vessels (guinea pigs and cats.) It diminishes the number of inspirations. It lowers the temperature uniformly in fatal cases. This lowering goes on to a greater and greater degree, to the end. In cases which recover, this period of lowering is succeeded by one of elevation of temperature, which returns to the original point, but in a longer time than that during which the abatement has operated.

Bromide of camphor possesses incontestable hypnotic qualities. Toleration is not established with this medicine, and its use gives rise, at least with guinea pigs, to a rapid, progressive emaciation.

